# Generating 3D architectured nature-inspired materials and granular media using diffusion models based on language cues

**DOI:** 10.1093/oxfmat/itac010

**Published:** 2022-11-11

**Authors:** Markus J Buehler

**Affiliations:** Laboratory for Atomistic and Molecular Mechanics (LAMM), Massachusetts Institute of Technology, 77 Massachusetts Avenue, Cambridge, MA 02139, USA; Center for Computational Science and Engineering, Schwarzman College of Computing, Massachusetts Institute of Technology, 77 Massachusetts Avenue, Cambridge, MA 02139, USA

**Keywords:** materials design, synthesis, architected material, hierarchical bio-inspired, language models

## Abstract

A variety of image generation methods have emerged in recent years, notably DALL-E 2, Imagen and Stable Diffusion. While they have been shown to be capable of producing photorealistic images from text prompts facilitated by generative diffusion models conditioned on language input, their capacity for materials design has not yet been explored. Here, we use a trained Stable Diffusion model and consider it as an experimental system, examining its capacity to generate novel material designs especially in the context of 3D material architectures. We demonstrate that this approach offers a paradigm to generate diverse material patterns and designs, using human-readable language as input, allowing us to explore a vast nature-inspired design portfolio for both novel architectured materials and granular media. We present a series of methods to translate 2D representations into 3D data, including movements through noise spaces via mixtures of text prompts, and image conditioning. We create physical samples using additive manufacturing and assess material properties of materials designed via a coarse-grained particle simulation approach. We present case studies using images as starting point for material generation; exemplified in two applications. First, a design for which we use Haeckel’s classic lithographic print of a diatom, which we amalgamate with a spider web. Second, a design that is based on the image of a flame, amalgamating it with a hybrid of a spider web and wood structures. These design approaches result in complex materials forming solids or granular liquid-like media that can ultimately be tuned to meet target demands.

## Introduction

Materials design using a variety of hierarchical architectures, including porous materials, has been a subject of intense research over the past decades [1–7]. While significant progress has been made, the exploration of novel architectures, and the use of unconventional sources as a body of knowledge to derive hierarchical structural designs, remains a challenge. One source for material design solutions is the use of biologically inspired paradigms, where a growing body of knowledge has contributed to new explorations in materials research [8–15].

Another avenue is the use of cross-cutting intersections of knowledge bases, integrating insights that incorporate a broad range of biological, human, cultural and scientific knowledge [16–18]. Such knowledge, collected across civilizations and eras of human development, and captured in the large body of knowledge encapsulated in the nexus of language, symbolism, images and associations between human thinking and physical or conceptual materializations of such, provides an important frontier in computationally driven design.

**Figure 1.**
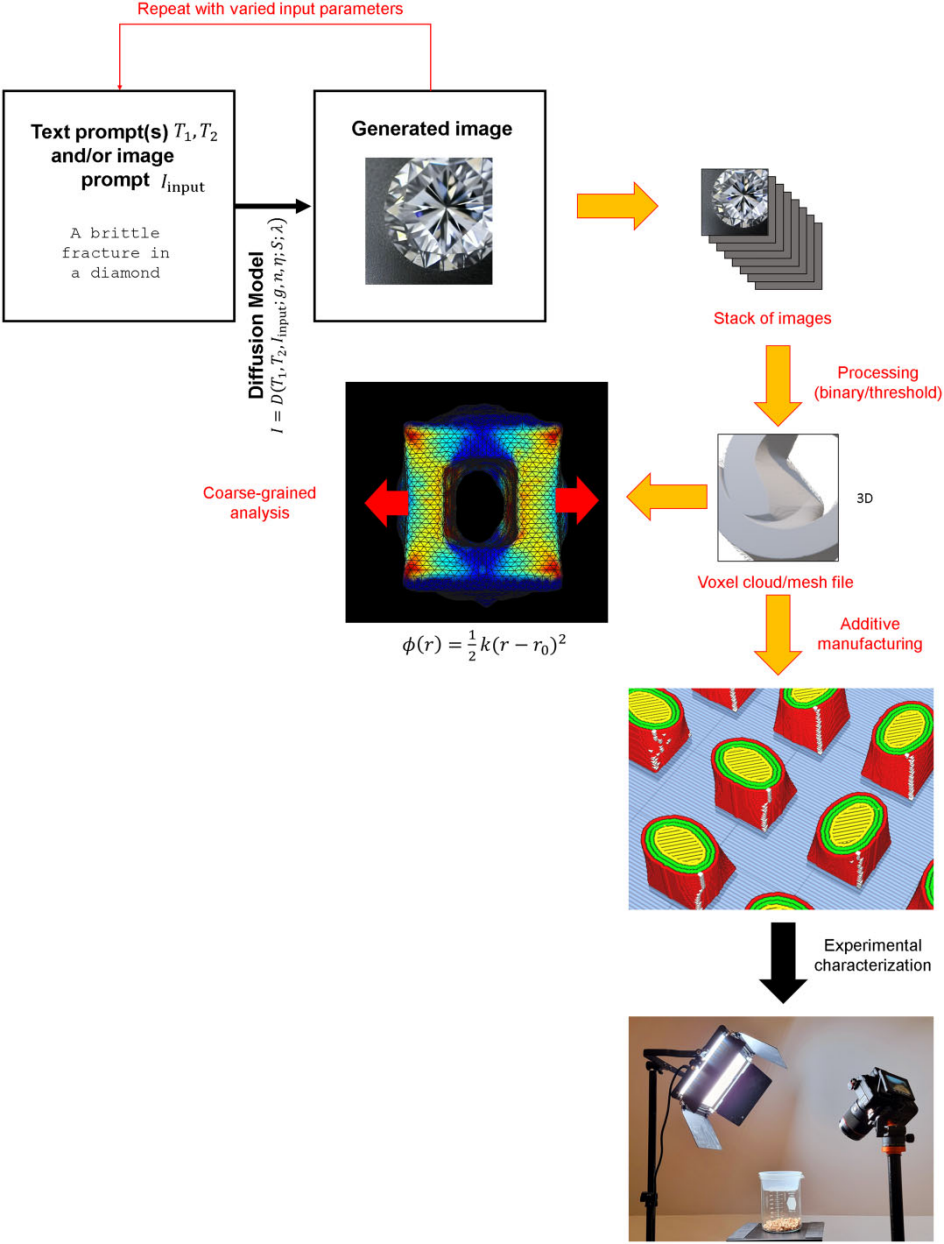
Schematic overview of the method developed and applied here. We start from text prompts, over which we interpolate in some form, resulting in a stack of images that form a voxel representation of a 3D material. These are then processed (here into black/white form, representing black = no material, white = material) and stacked, forming a voxel representation that is also translated into a 3D mesh. The mesh can be used either for simulation analysis or for additive manufacturing, followed by experimental assessment.

To solve such problems, the use of deep learning offers avenues toward fundamental solutions to these challenges. Recently, a variety of image generation methods have been proposed, notably DALL-E 2 [19], Imagen [20] and Stable Diffusion [21]. In this article, we focus on a specific aspect, to explore to what extent these methods can be used for broader sets of Nature-inspired materials design applications [22–27], realizing the overall approach summarized in Fig. 1. In earlier research [28–31], text-to-material translations have already been examined, including using combinations of CLIP with VQGAN [32]. This enabled not only the translation of text to material designs, based on comprehensive training data that represent a broad spectrum of all vision–text pairs created through media collections like the Internet, but also enabled researchers to direct assembly of custom material building blocks into specific shapes and patterns, such as done for the case of flame particles [28].

### Objectives of this study

A key objective of this study is to explore the cross-pollination of different fields; and specifically, how information from one modality of context can be translated into another, through rigorous categorization [16, 17, 33–35]. Here, we focus on translations from the corpus of language-image pairings as captured in Stable Diffusion, toward 3D material design explorations. We are specifically interested to explore bio-inspired design cues, where we seek to translate patterns found in natural systems such as spider webs [36–39], wood microstructures [40–42] or protein patterns [33, 43, 44] into innovative material architectures, building on earlier bio-inspired design work [8–15].

Here, we build on this work, but use more sophisticated image generation tools, using diffusion architectures [20, 21, 45–50] (Buehler, unpublished work [51]), and a fundamentally different approach for text generation where synthesis no longer requires an iterative examination of latent space through co-operation with a CLIP classifier, but that facilitates synthesis of images directly from a seed noise vector. This approach provides numerous advantages, including much higher resolutions and image fidelity, which as is shown in this article bodes well for material design applications. Moreover, models like Stable Diffusion or DALL-E 2 [19, 21, 52] emerge as a representation of a broader collective human corpus of visual–text pairings, which can be a powerful reservoir for materials design applications. In the spirit of what is referred to as bio-inspired design, this approach represents a variation of the concept to translate design ideas across modalities and physical realizations.

The general mathematical context of these models is that they produce an image I from a given text input T (and an optional image conditioning, $I_{\mathrm{input}}\;$used during image-to-image translation), as well as a set of synthesis parameters p:

(1) $$I=D(T,I_{\mathrm{input}};p).$$

The text prompt is simply provided as string data and p includes synthesis parameters g (guidance scale in text-to-image generation or strength parameter within 0…1 in image-to-image generation guided by text), n (number of inference steps), η (between 0.1, where lower values typically yield better quality, higher values more diverse results), combined with a random seed S (implemented via a PyTorch global seed):

(2) p=(g,n,η;S).

The parameter g delineates how strongly the model follows the text prompt, as opposed to generating more random solutions. Altogether, the image generation model can be mathematically summarized to yield images I based on its salient input parameters as

(3) $$I=D(T,I_{\mathrm{input}};g,n,\eta ;S).$$

The standard resolution of images generated with Stable Diffusion is 512 × 512, albeit the method can also generate larger-resolution images (unless otherwise indicated, we use the standard resolution in image synthesis; Supplementary Fig. S1 shows examples for images generated at 512 × 512 and 1024 × 1024 resolution).

A challenge that needs to be overcome is that materials typically require 3D realizations; quite distinct from 2D image data. While there has been work on 3D model generation [53], sophisticated text-to-image diffusion models cannot yet generate 3D representations. Other methods that have been proposed is to use neural networks to predict 3D data directly from 2D visuals [54]; however, these tools are relatively early in their development stages and not generally applicable. Hence, we set forth a simple but structurally diverse and rich algorithm that enables the direct use of state-of-the-art image generation tools, specifically Stable Diffusion [21] (but the method could generally be applied to other methods as well including for novel neural network architectures that are trained specifically with this downstream application in mind). We further outline a method to rapidly assess properties of the generated design, realized using a coarse-grained particle model that offers a means to assess mechanical, vibrational or other features. While this is beyond the scope of this initial article, future work could use this pipeline as a way to conduct a broad search of the design space defined by the nexus of the lexicon of human language, knowledge and mathematically or statistically parameterized, or learned, latent space.

## Methods

### Generative model: interpolating and mixing text conditioning

The method used here is based on a pre-trained Stable Diffusion model and using the sd-v1-4.ckpt [55] weights. We create a variety of functions that change the way by which images are synthesized, enabling multiple text prompts and an iteration between such text prompts using a weighting function. In the Stable Diffusion model, a text prompt T is translated into an embedding tensor E that captures the coding of the particular input provided in a form that the generative diffusion model understands as a conditioning to produce a particular image that reflects the text.

Building on this approach, in order to use multiple text prompts, we first generate embeddings $E_{1}$ and $E_{2}\;$for two text prompts $T_{1}$ and $T_{2\;}$ provided, following generally $E_{i}=f_{\mathrm{text}\;\mathrm{emb}}(T_{i})$. The two embedddings are then mixed according to a weight λ (between 0 … 1):

(4) $${E=\lambda E}_{1}{+(1-\lambda )E}_{2}.$$

This then results in an expanded generator model that features multiple text prompts and the weight parameter λ:

(5) $$I=D(T_{1},T_{2},I_{\mathrm{input}};g,n,\eta ;S;\lambda ).$$

Before translation to 3D representation, the images I generated by the diffusion model are processed. In this study, we focus on processing to convert shades of color or intensity into a binary representation, primarily because we aim to design and manufacture materials with two material types only: void, and material present, at each point in 3D.

First, we translate the image into a binary B&W representation using cv2.threshold. We resize the image to the desired output resolution, and apply cv2.bilateralFilter and cv2.GaussianBlur, followed by a second cv2.threshold operation. This helps to generate smooth contours, of white (material) and black (no material) distributions. Next, we remove small white areas from each image to avoid too many small clusters that can be difficult to manufacture. We achieve this by finding all contours using cv2.findContour, and then remove areas below a certain threshold by drawing over with the black signal. The approach could easily be modified to use other transformations, for example, realizing multiple-material outputs depending on color or texture produced by the generative model.

Within the set of parameters p, we typically use g=7.5 and n = 20. Random seeds are utilized to generate images from noise vectors and controlling the seeds enables us to reproduce results deterministically.

### Generating a voxel representation in 3D and translation to a mesh model

**Figure 2.**
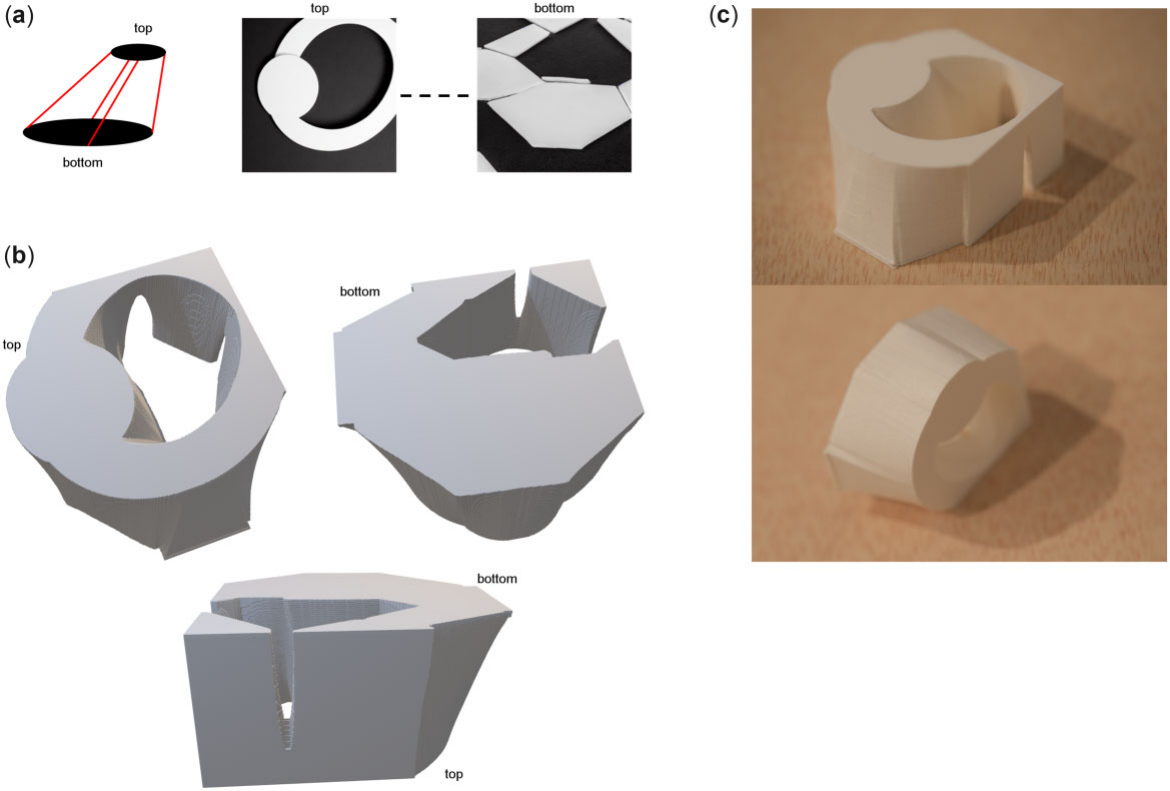
Using two prompts as input, and smoothly interpolating between these two designs to generate a larger voxel representation. (**a**) Left: Illustration how we interpolate between two source images, generating a number of intermediate layers, resulting in a voxel grid that captures the initial 3D geometry produced. Right: The two source images generated from the prompts $T_{1}$ = ‘a small white circle on black background’ and $T_{2}$ = ‘a large white hexagon with sharp edges on black background’. Parameters used are p=(g=0.8,n=20;S=343 613). (**b**) Resulting 3D geometry viewed from different angles. (**c**) Physical samples manufactured using 3D printing, shown from a few angles to visualize the final results.

In order to generate 3D architectures, we introduce a voxel representation. As a basic step, each image is translated into one sheet of voxel as described above. We either use the diffusion model to generate a series of images and from them, voxel sheets, that are stacked together to form a 3D representation of material, or use a small set of voxel sheets and generate interpolations between them. In the interpolation case, we use scipy.interpn to interpolate between two contours, resulting in a smooth transition between the top and bottom voxel sheets (for a schematic of how this process works, see example in Fig. 2a, left).

For applications in analysis (coarse-grained modeling) or additive manufacturing, the voxel data are translated into a 3D mesh representation using trimesh. For this step, the voxel stacks are processed using the trimesh.voxel.ops.matrix_to_marching_cubes function. As an option, the resulting meshes are smoothed using trimesh.smoothing.filter_mut_dif_laplacian or used directly, as predicted.

### Coarse-grained model for mechanical assessment

To illustrate the potential to examine physical properties of the generated structures, we implement a coarse-grained LAMMPS model [56]. We consider the mesh file (e.g. loaded as STL file) and insert a regular face-centered cubic particle structure in the inside of the mesh (alternatively, we can work directly with the voxel data but using mesh data as input enables users to potentially process the mesh files in other code or combine multiple mesh files into larger assemblies). Each particle interacts according to a harmonic inter-particle energy potential, defined as

(6) $$\phi \left(r\right)=\frac{1}{2}k{\left(r-r_{0}\right)}^{2}.$$

We choose k=1.5 and $r_{0}=1.0$. Only nearest neighbor particles interact. Bonds can never break during simulations, as defined by the interparticle energy model in Equation (2).

We implement displacement boundary conditions, where the top and bottom row of the particle system are fixed and move according to a prescribed pulling rate to implement mechanical deformation.

The resulting data are analyzed using Python scripts and visualized using OVITO [57]. We use mesh representations of the particle model to visualize the 3D structures, with color codes to indicate the stress level (blue = low stress and red = high stress).

### Additive manufacturing

We use a Ultimaker S3 fused deposition modeling (FDM) 3D printer with white polylactic acid (PLA) filament to produce the physical samples. The granular media are produced using a QIDI PRO 3D printer with wood-based PLA filament, hence the wood-like color visible in the photographs. Supplementary Movie M6 shows a video of the additive manufacturing process for this and some of other samples reported in this article.

## Results

The purpose of this article is to report the general methodology and to implement a first demonstration of the proposed concept. We cover both, generation of 3D architectures from various text prompts, as well as generating a new form of granular media by generating a large ensemble of text generated particles.

**Figure 3.**
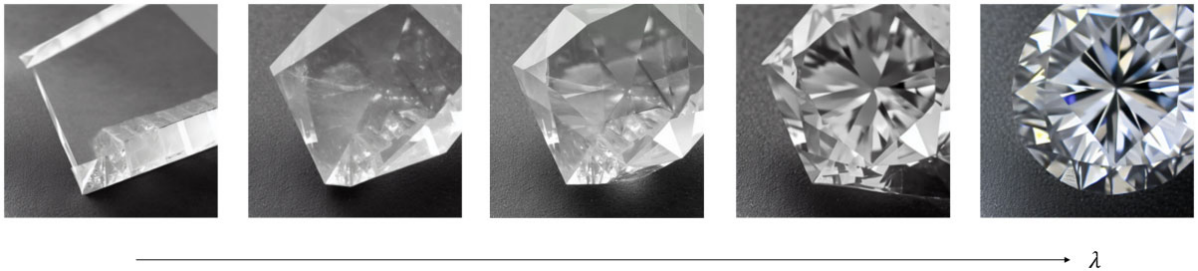
Interpolation between two text prompts by varying λ, resulting in continually varying image output. The left corresponds to $T_{1}$ = ‘a fracture in glass with sharp edges’ (left) and the right to $T_{2}$ = ‘a brittle fracture in a diamond’ (right), with smooth interpolation between. Supplementary Movie M1 shows an animation across all generated 512 frames. Parameters used are p=(g=0.75,n=40;S=343 613).

First, we demonstrate the use of the mixed text embeddings in generating continuously varying images, as shown in Fig. 3. We use

$T_{1}=$ ‘a fracture in glass with sharp edges’

$T_{2}=$ ‘a brittle fracture in a diamond’

and vary λ from 0 … 1 in 512 steps, generating 512 frames that are then rendered as a movie (Supplementary Movie M1).

This example already shows an interesting transition between the two prompts; in early stages, the generated image reflects that of prompt $T_{1}\;$whereas at the end, it approaches the solution of $T_{2}$. The transition between the two is not ‘linear’ but rather induces more sudden transitions in the type of generated images. We use this example as motivation to generate complex, more abstract designs by mixing individual prompts.

Figure 3 depicts results for two distinct prompts as input, and smoothly interpolating between them to generate a larger voxel representation of a 3D engineering design. Going into details of the process, Fig. 3a shows the two source images generated from the prompts $T_{1}$ = ‘a small white circle on black background’ and $T_{2}$ = ‘a large white hexagon with sharp edges on black background’. Figure 3b shows the resulting 3D geometry viewed from different angles. Finally, Fig. 3c shows physical samples manufactured using 3D printing.

**Figure 4.**
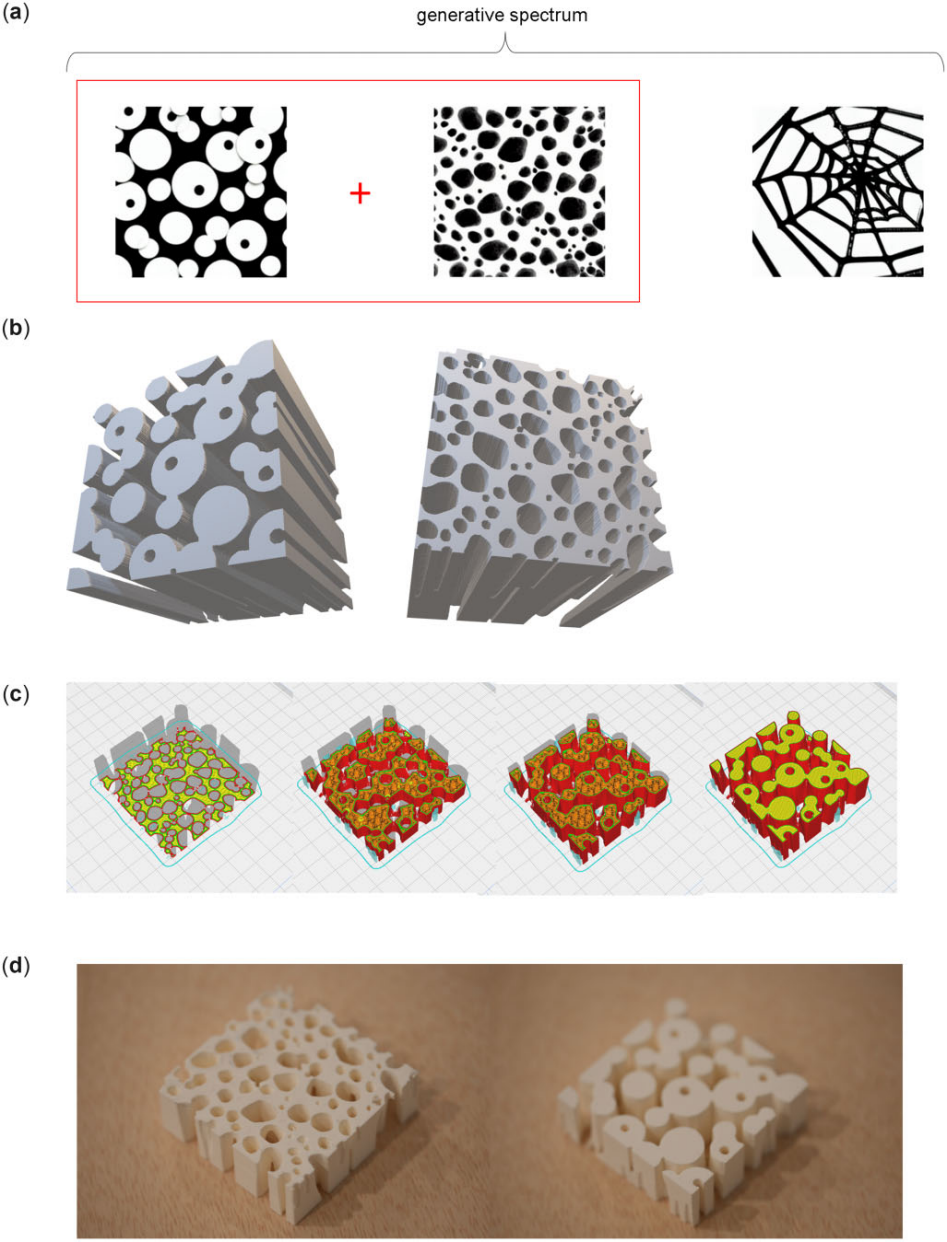
Generation of architectured materials from diffusion models. We use two text prompts $T_{1}$= ‘several small white circles on black background’ and $T_{2}$ = ‘a large triangle in the shape of a spider web on black background’ and interpolate in three steps between them. Parameters used are p=(g=0.8,n=20;S=33). (**a**) Depiction of the three designs generated; however, only the left and center design are used for interpolation. It is seen that the mixing of the two prompts at λ = 0.5 (the center result) yields an interesting design that neither text prompt alone could have generated. (**b**) Visualization of the resulting 3D geometry from two angles, from top/bottom to show the distinct features at either end. (**c**) 3D printing process, here showing the slicing of the structure and generation of an internal gyroid structure. (**d**) Final 3D printed sample of the material.

Next, we explore the potential to use a mixing of text prompts as a way to yield interesting designs, using λ. Figure 4 depicts the results of generation of architectured materials [58] through interpolation, by varying the λ parameter in Equation (5). We use two text prompts $T_{1}$=‘several small white circles on black background’, and $T_{2}$ = ‘a large triangle in the shape of a spider web on black background’ and interpolate in three steps between them. Figure 4a shows a depiction of the three designs generated. We find that the left and center design—where the center design reflects a particularly interesting outcome, achieved by mixing the two prompts at λ = 0.5. Figure 4b shows a visualization of the resulting 3D geometry from two angles, from top/bottom to show the distinct features at either end. To illustrate the process by which the design is manufactured, Fig. 4c shows details of the slicing in the 3D printing process (we use an internal gyroid structure). Figure 4d then depicts the final 3D printed sample of the material.

**Figure 5.**
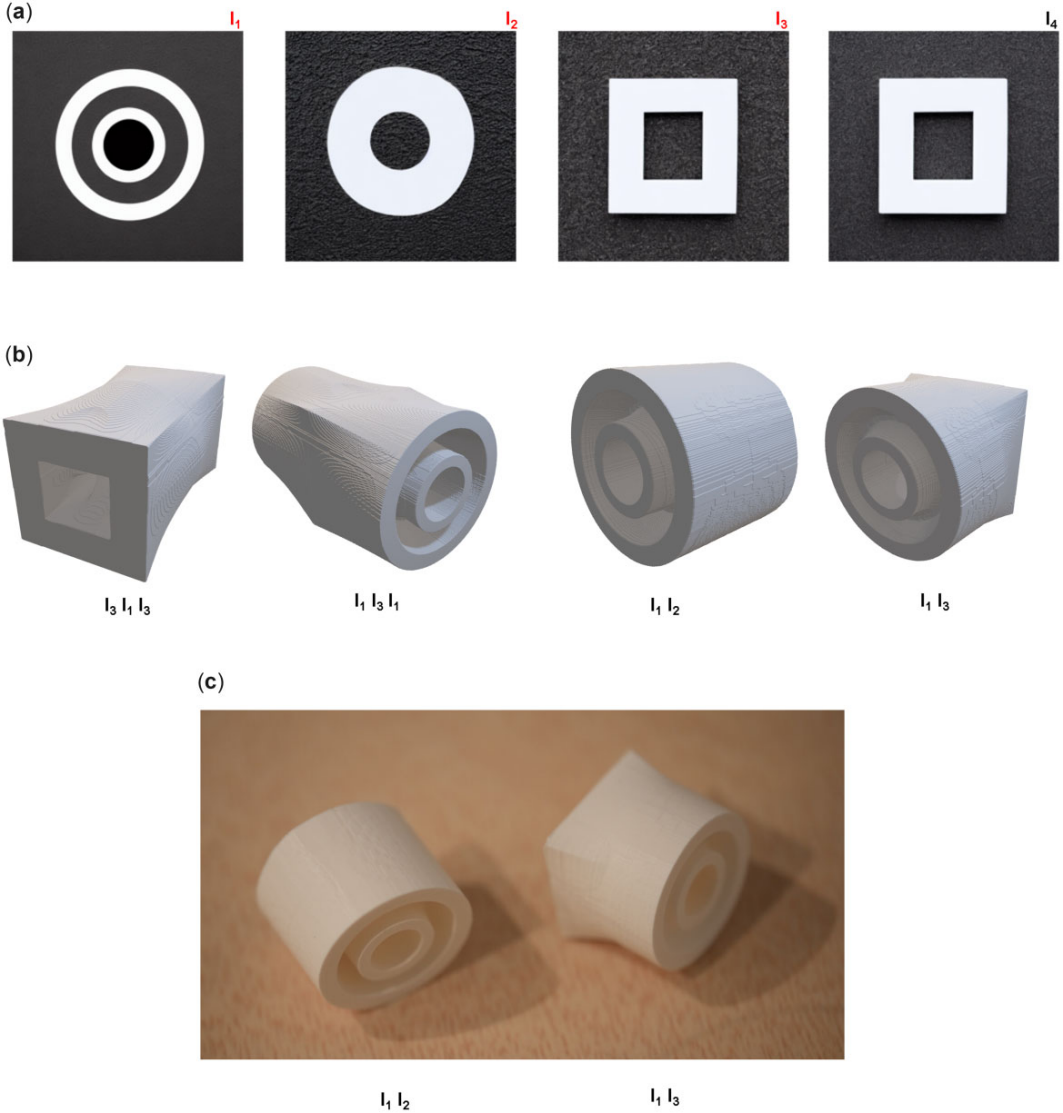
Various designs from simple original input, assembled in different organizations. The original generation results in four distinct designs I_3_, I_1_, I_3_ and I_4_ (since I_3_ ≈ I_4_, we only use the first three in the design process). We use two text prompts $T_{1}$ = ‘a white circle on black background’ and $T_{2}$ = ‘one large white square centered on black background’ and interpolate in four steps between them. Parameters used are p=(g=0.75,n=30;S=3433). (**a**) Original image results I_*i*_ before processing. (**b**) Various permutations of the elemental designs and resulting 3D structures. (**c**) Results of 3D printed samples, for two of the designs.

The image generation model with its various parameters delineated in Equation (5) can be used to define a set of building blocks that can be arbitrarily combined to yield interesting combinatorial options. Figure 5 realizes this idea and presents designs from simple original input, assembled in different organizations. The original generation results in four distinct designs I_3_, I_1_, I_3_ and I_4_ (since I_3_ ≈ I_4_, we only use the first three in the design process). As input we use two simple text prompts $T_{1}$ = ‘a white circle on black background’ and $T_{2}$ = ‘one large white square centered on black background’ and interpolate in four steps between them. Figure 5a shows the original Image results I_*i*_ before processing. Figure 5b then shows various permutations of the elemental designs and resulting 3D structures. Figure 5c displays photographs of 3D printed samples, for two of the designs.

**Figure 6.**
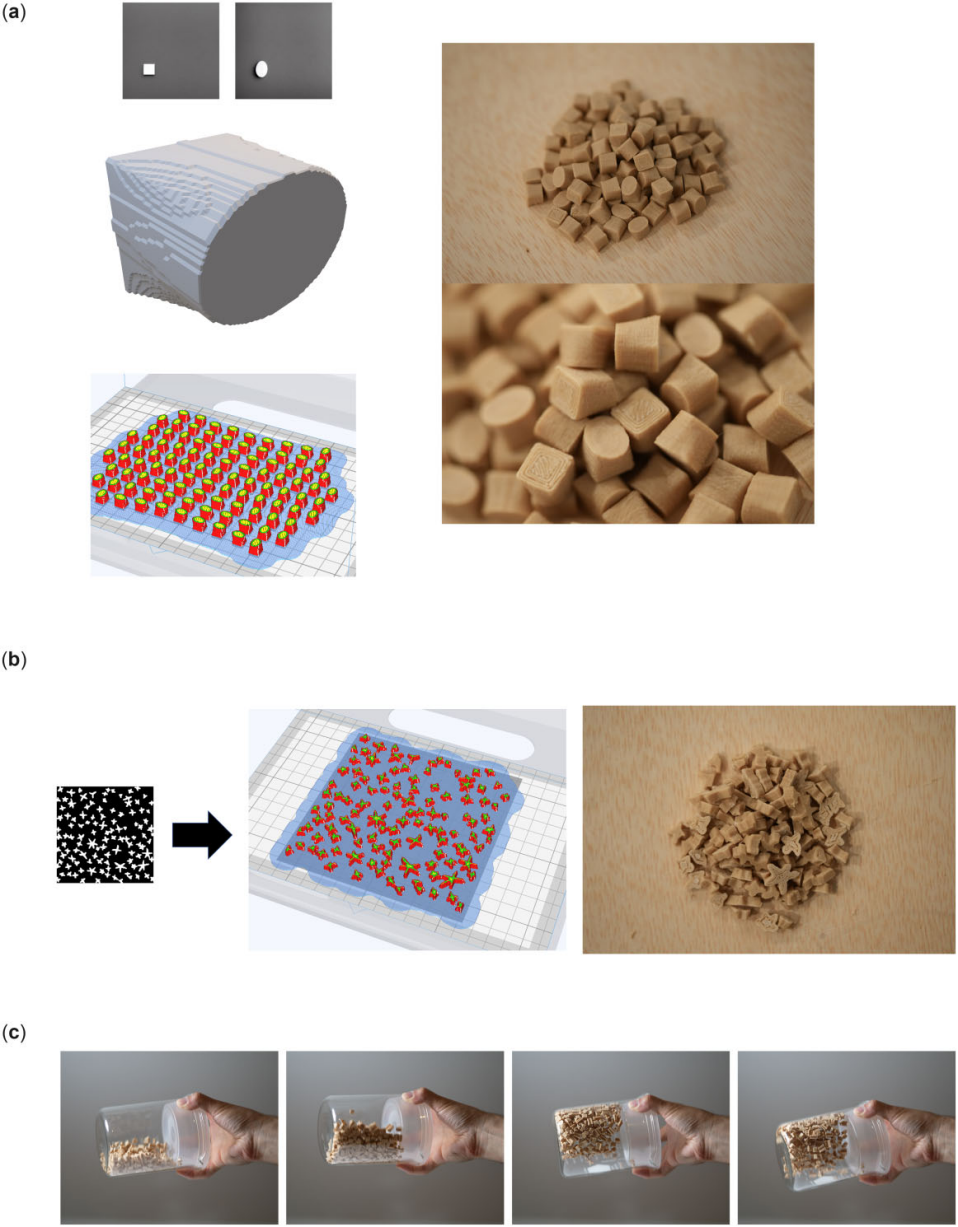
Generation of granular media using the approach, for different design cues. (**a**) Text prompts $T_{1}$ = ‘a white circle on black background’ and $T_{2}$ = ‘a white oval on black background’. Parameters used are p=(g=7.5,n=40;S=3431), with a resolution of 1024 × 1024. This generation task results in a single particle; it is replicated *N* times and then 3D printed, forming the granular material shown on the right. (**b**) The design is generated using a single text prompt, *T* = ‘white bright stars in the shape of a spiral on black background’ and then made into a 3D design by extrusion in 3D paint. Parameters used are p=(g=5.5,n=60;S=34 353 531), with a resolution of 1024 × 1024. We generate two sets of these star-shaped granules with different height (3.3 mm and 10.5 mm). (**c**) Simple manual shaking experiment conducted to demonstrate the liquid-like mechanical behavior of the resulting material. Supplementary Movies M2–M4 show additional experiments, recorded in slow-motion.

The method cannot just be used to make singular architectural materials. Using additive manufacturing, we can easily generate hundreds of copies of particles, each of which is designed using the diffusion approach described here. Figure 6 demonstrates this granular media generation approach, for different design cues. In the design used in Fig. 6a, we trigger synthesis using text prompts $T_{1}$ = ‘a white circle on black background’ and $T_{2}$ = ‘a white oval on black background’. As can be seen, this generation task results in a single particle. The singular particle is then replicated a larger number of times and then 3D printed, forming the granular material shown on the right. Figure 6b shows how many variations of a design can be generated using a single text prompt, *T* = ‘white bright stars in the shape of a spiral on black background’. Since this prompt results in many particles at the same time, we can directly produce a granular material from the resulting image. We generate two sets of these star-shaped granules with different height using a simple extrusion approach. Figure 6c shows the result of a simple shaking experiment conducted to demonstrate the liquid-like mechanical behavior of the resulting material. Supplementary Movies M2–M4 show additional shaking experiments, recorded in slow-motion, to demonstrate the characteristic of the produced material as a fluid-like substance.

**Figure 7.**
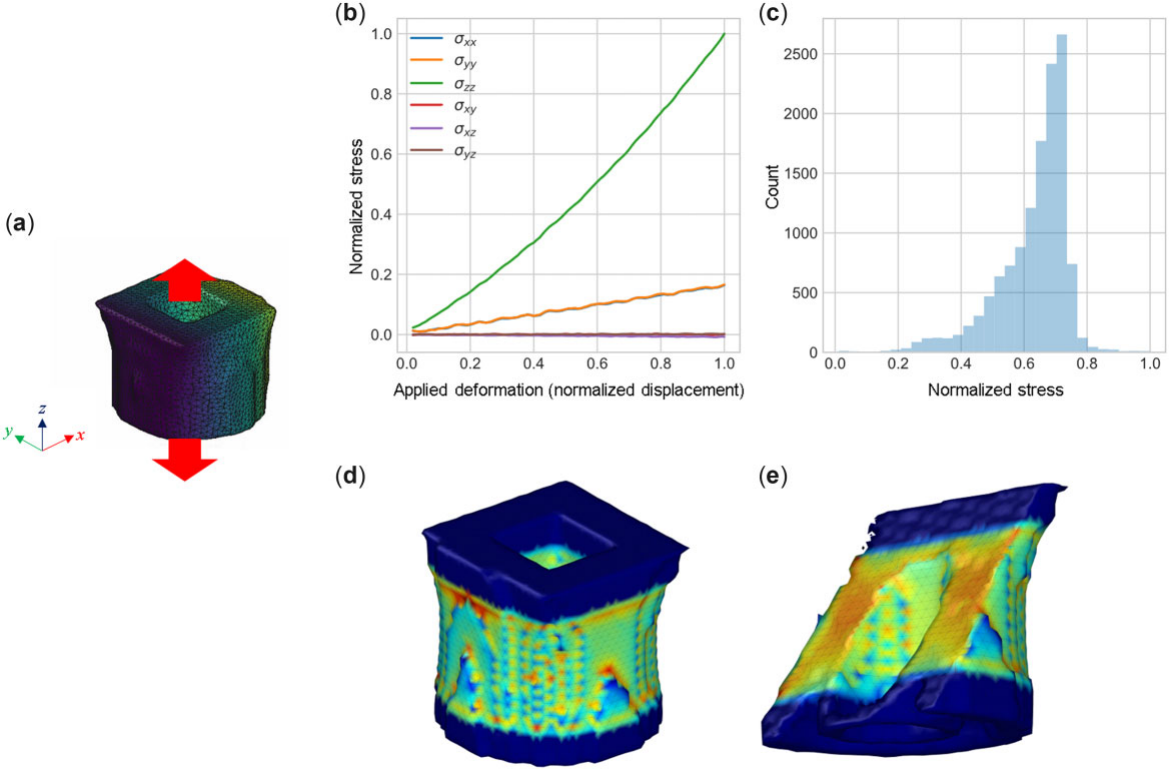
Mechanical assessment of one of the designs shown in Fig. 5. We limit the exploration to a simple tensile test (**a**), resulting in stress–strain curves (**b**), a depiction of the Von Mises stress (**c**), and a stress field in the 3D domain (**d**) and a cross-section (**e**). All stresses and displacements are plotted in non-dimensional units, normalized by the largest stress/displacement in the numerical experiment.

While we limit ourselves largely to exploring the generation process and how various parameters affect the result, future work should focus on characterizing the designs to meet certain objective demands. For this scenario, either experimental or computational assessment methods are needed. Figure 7 shows a simple method to offer a rigorous mechanical assessment of one of the designs shown in Fig. 5. We limit the exploration to a simple tensile test (Fig. 7a), resulting in stress–strain curves (Fig. 7b), a depiction of the Von Mises stress (Fig. 7c), and a stress field in the 3D domain (Fig. 7d) and a visualization of the internal stresses via a cross-sectional view (Fig. 7e). The results depicted here are based on a coarse-grained model that captures, via a shape-based mesoscale model, elementary structure–function relationship. Developed directly based on the geometry file produced by the generative method (from an STL file), the simulation approach allows us to explore various mechanical boundary conditions, including a tensile test as done here. The data produced by such a method can easily be integrated with a Bayesian optimization algorithm, and parameters in Equation (5) could be tuned to meet certain design demands.

**Figure 8.**
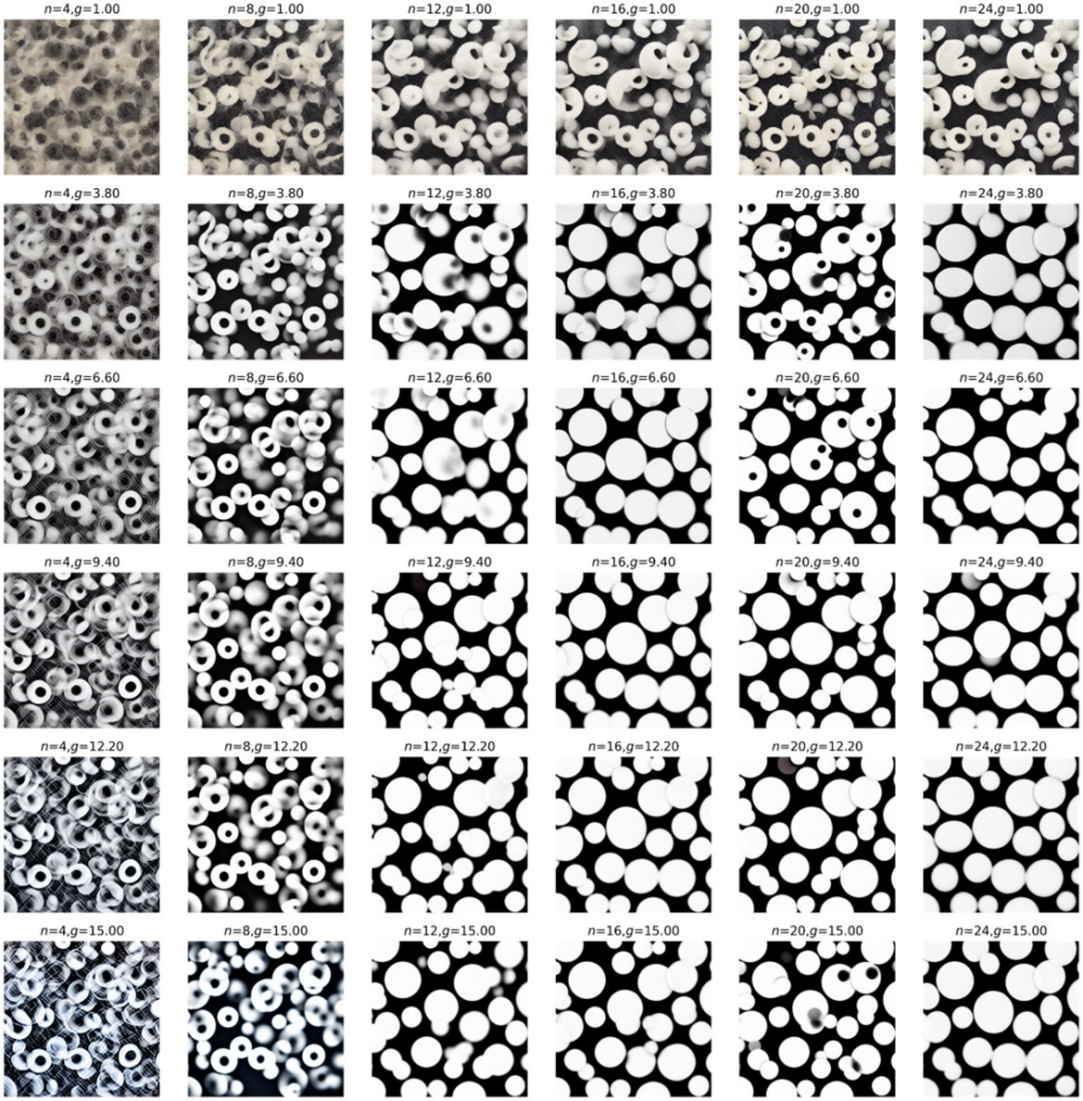
Systematic variations of parameters inference steps *n* and guidance scale *g*. Text prompt T = ‘several small white circles on black background’. Other constant parameters p=(S=33).

**Figure 9.**
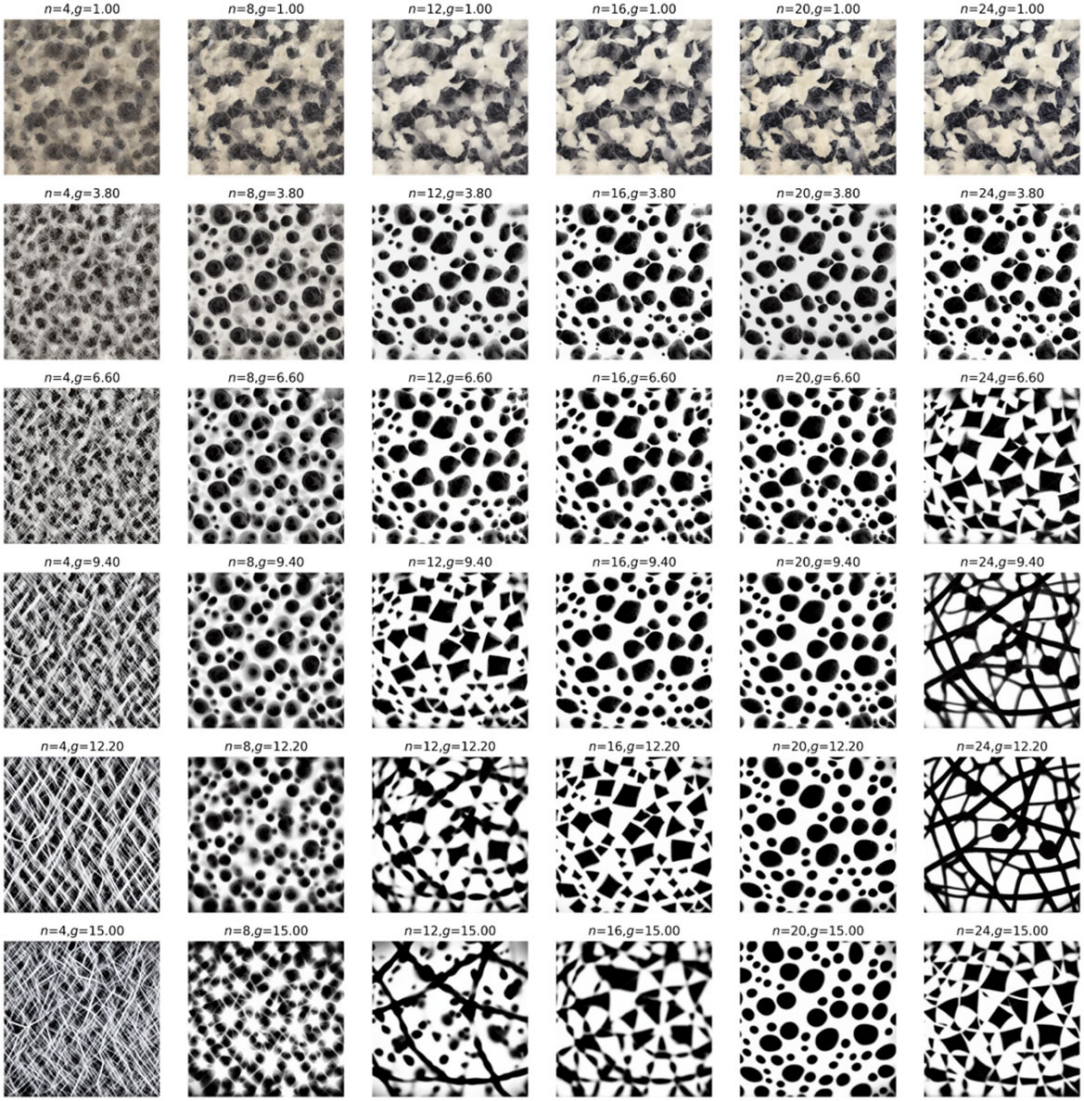
Systematic variations of parameters inference steps *n* and guidance scale *g*. Text prompts are $T_{1}$ = ‘several small white circles on black background’ and $T_{2}$ = ‘a large triangle in the shape of a spider web on black background’. Other constant parameters p=(S=33, λ=0.5).

We now show a couple of systematic explorations of key parameters in Equation (5) and examine how they affect the produced images, and by extension, how they affect the design cues we can utilize for 3D material construction. Figure 8 displays the results of systematic variations of inference steps *n* and guidance scale *g*. Similarly, Fig. 9 shows results of a systematic variation of inference steps *n* and guidance scale *g*, but this time for two text prompts $T_{1}$ = ‘several small white circles on black background’ and $T_{2}$ = ‘a large triangle in the shape of a spider web on black background’ mixed with λ=0.5.

**Figure 10.**
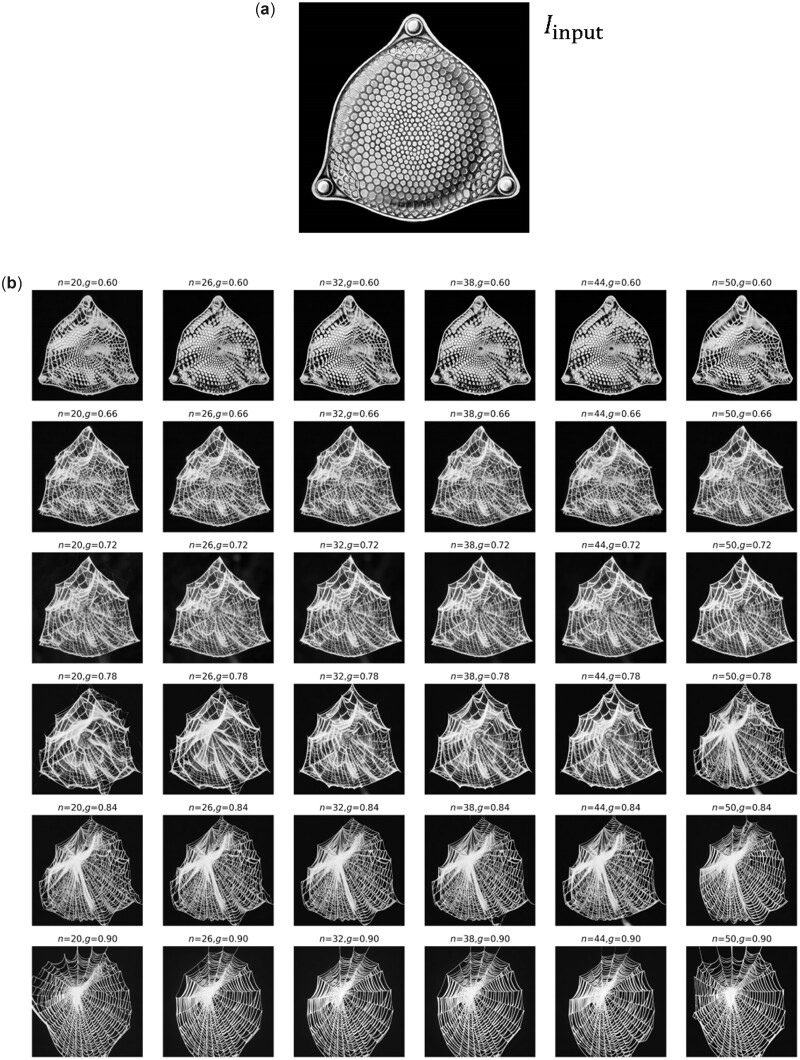
Generation of a variety of images, based on a starting image $I_{\mathrm{input}}$—a diatom structure (**a**), based on Haeckel’s lithographic print of a diatom [59]—for variations of parameters inference strength *g* and *n*. The text prompt is $T_{1}$ = ‘a spider web with thick white lines on black background’. Other constant parameters p=(S=33), resulting in the images summarized in (**b**).

**Figure 11.**
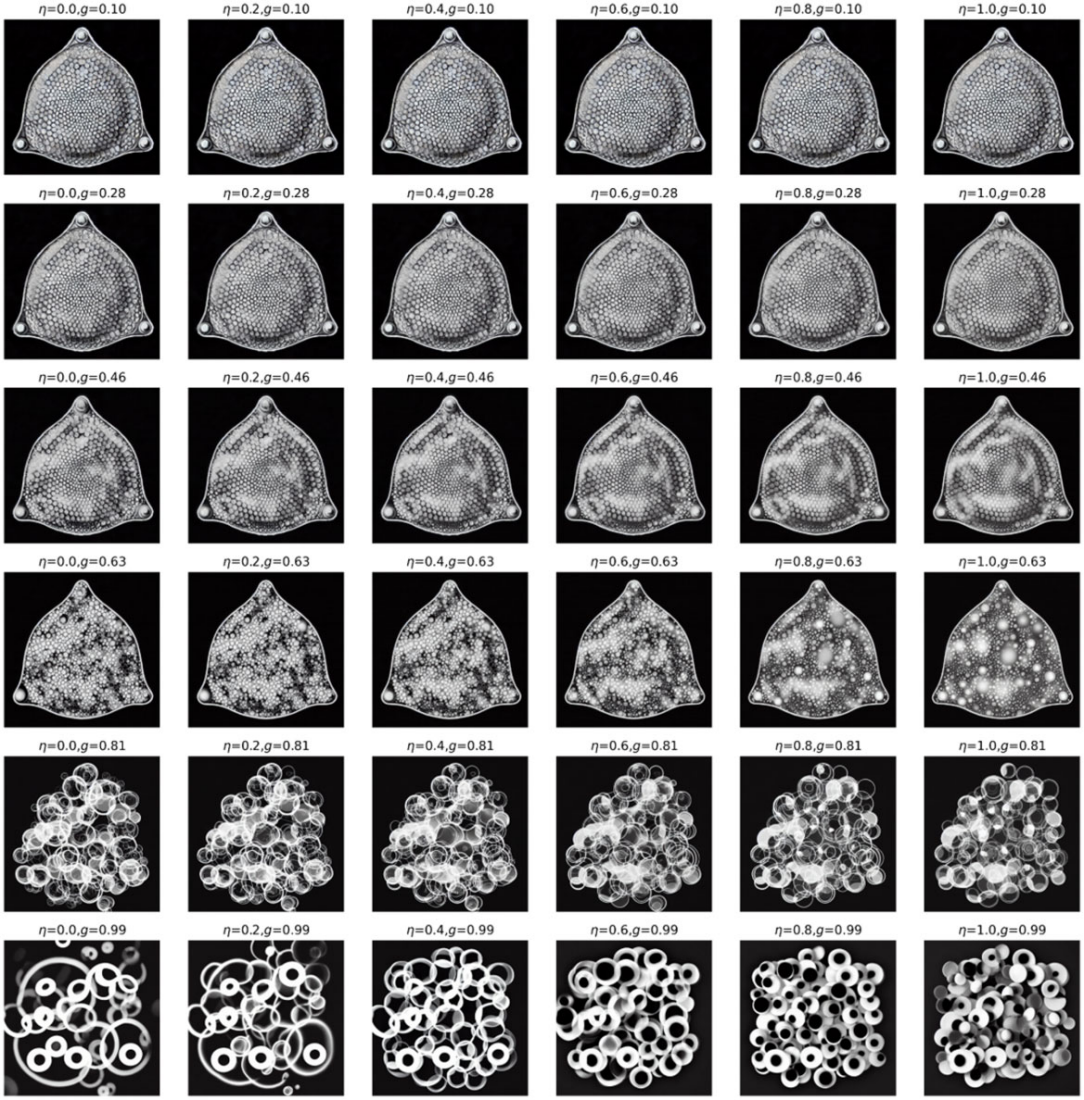
Variation of η and strength *g*. The text prompt is $T_{1}$ = ‘several small white circles on black background’. Other constant parameters p=(S=33).

**Figure 12.**
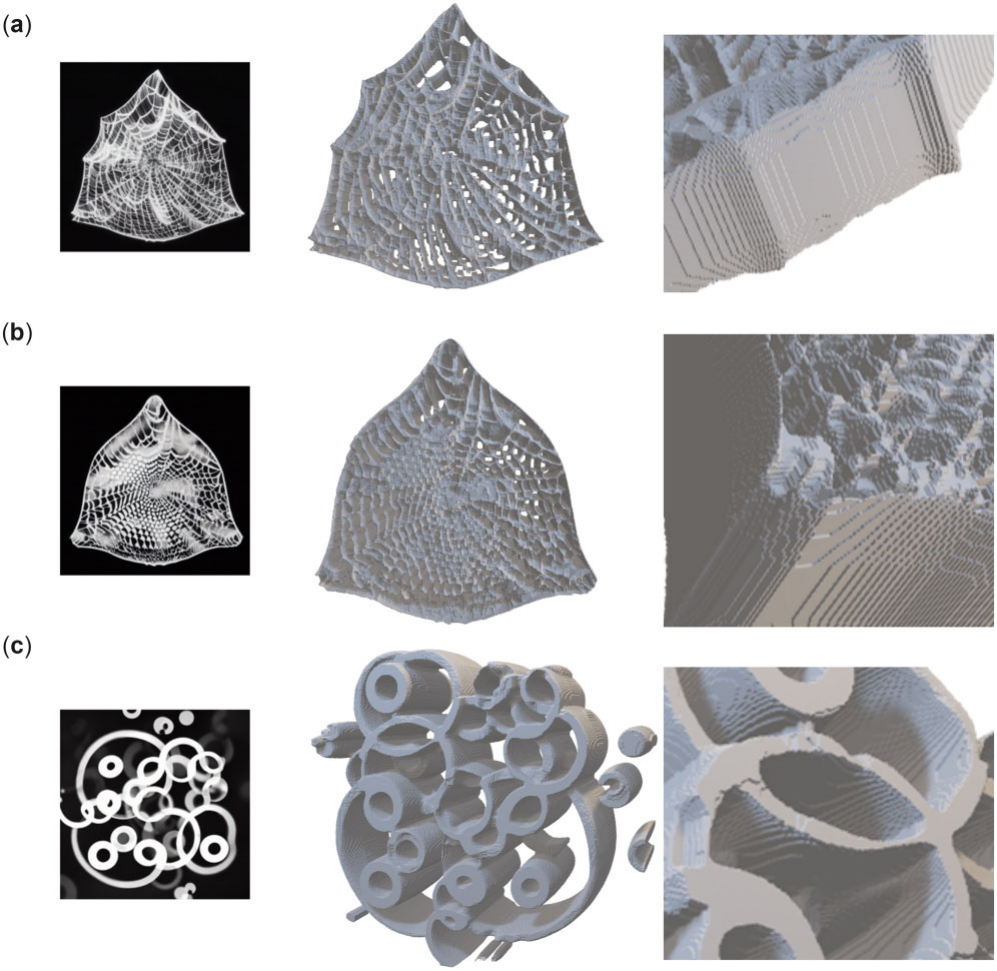
Translation of the hybrid diatom-spider web structures (shown in Figs 10 and 11, and Supplementary Fig. S2) into a 3D architecture, for three different examples shown in panels (a), (b) and (c). For these examples, we use the pixel color intensity to map to depth of the resulting 3D structure; bright/white = maximum height, dark/black = no material. This method is applied symmetrically in both out-of-plane directions (forward and backward) to yield a 3D material architecture.

Thus far, all image generation tasks were conducted solely based on text prompts (either a single one or mixed prompts to enhance design diversity). Now, we use also an input image to condition the generation, in addition to one or more text prompts. As will be shown, this offers a tremendous range of controllability and expressiveness in terms of inducing highly complex design concepts that cross or amalgamate cues provided. Figure 10 shows the results of these computational experiments, depicting generation of a variety of images based on a starting image as an additional input. In this case, we use a diatom structure as input image (Fig. 10a) for variations of parameters inference strength *g* and *n*. The diatom image is extracted from Haeckel’s lithographic print of a diatom from *Kunstformen der Natur* (English: *Art Forms in Nature*), as reported in Ref. [59]. The text prompt is $T_{}$= ‘a spider web with thick white lines on black background’. Results are shown in Fig. 10b. The palette of designs can then serve as a starting point for further exploration or can be combined into a set of material building blocks akin to what was presented in Fig. 5. By playing with the text prompt, one can achieve very distinct material design outputs. Figure 11 shows a variation of η and strength *g* (all while using the same image prompt as input as shown in Fig. 10a). The text prompt used here is $T_{}$ = ‘several small white circles on black background’. Another example for different parameter variations is shown in Supplementary Fig. S2. These broad variations in design can be turned into 3D architecture materials, as shown in Fig. 12 for a few examples.

**Figure 13.**
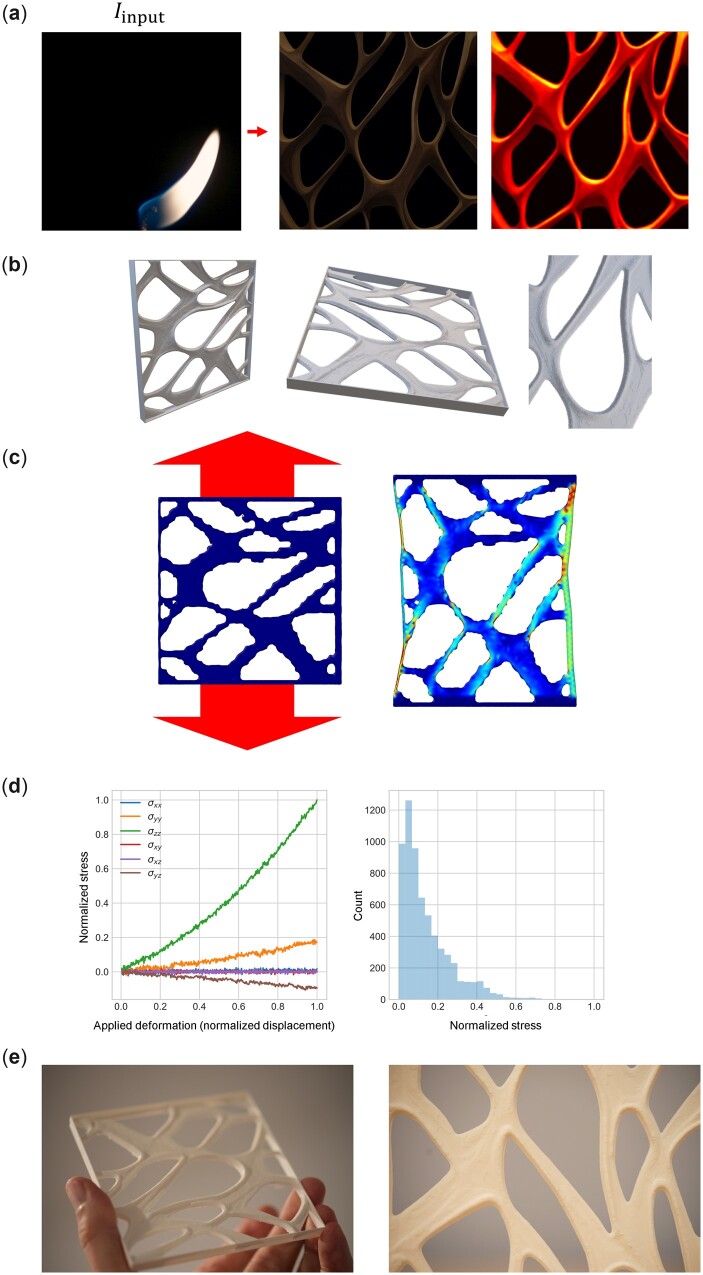
Entire workflow from design to modeling to manufacturing, for a design based on the intersection of a flame image with a complex text prompt featuring are $T_{1}$ = ‘a spider web with thick lines on black background’ and $T_{2}$ = ‘the internal details of wood microstructure’. Other constant parameters p=(S=33, λ=0.25). (**a**) Overview of the design steps from raw image to symmetrically extruded 3D material with a box added at the exterior as shown in (**b**) (we follow the same process of symmetrically extruding the image based on pixel intensity as explained in Fig. 12). Panel (**c**) shows a simple mechanical assessment under tensile deformation and (**d**) shows the resulting stress–strain results and stress field statistics for the Von Mises stress. (**e**) Photographs of the final manufactured material using FDM 3D printing. Supplementary Movie M^4^ depicts the stress field as the sample is deformed. Supplementary Movie M^6^ shows a recording of the additive manufacturing process for this and some of other samples reported in this article.

By controlling the input image, we can expand the space of resulting nature-inspired designs. For instance, Supplementary Fig. S3 shows a candle-based design, where text prompts are $T_{1}$ = ‘a spider web with thick lines on black background’ and $T_{2}$ = ‘the internal details of wood microstructure’, with λ=0.25.Figure 13 presents the entire workflow from design to modeling to manufacturing, for a design based on the intersection of a flame image with such a complex text prompt, for one of the designs generated (see red mark in Supplementary Fig. S3 for the one picked). Supplementary Movie M^5^ shows a movie of the tensile deformation simulation of the material. The results reveal that interesting material designs can be generated from a rich repertoire of design ideas, accessed directly via a combination of human language input, nature-based design ideas and mathematical parameterization. The resulting architecture material combines features from all of these foundational cues and amalgamates them into an intricate structural design. The mechanical assessment, following a similar approach as done earlier for the results in Fig. 7, provides us with a quantitative mechanism to better understand the design, score key performance measures and optimize the resulting designs to meet a set of demands.

## Discussion and conclusion

In this article, we used a pre-trained Stable Diffusion model and consider it as an experimental system—reflecting a broad corpus of human knowledge—to examine its capacity to generate novel material designs specifically in the context of 3D material architectures. Such materials design may find many applications ranging from optical to mechanical [60, 61] or multifunctional and integrated responsive material systems [62, 63].

We demonstrated that this approach offers a useful paradigm to generate a variety of novel material designs, using human language as a reservoir for cultural and civilization-spanning knowledge as design input, and exploring a vast nature-inspired portfolio of architectures and patterns. We present a series of methods to translate 2D representations into 3D data, including movements through noise spaces, mixtures of text prompts and interpolations. We manufactured several samples using additive manufacturing [15, 64, 65], and presented a method to assess the mechanical features (including stability) and other structural material properties of materials designed in that way, based on a coarse-grained shape-based particle simulation.

Specific objective functions that score material designs for alternative target properties, beyond mechanical stability, could be developed, based on existing literature. For instance, optimization work to design photonic crystal structures has been reported [66, 67], which could be enriched with the tools described here. Within this context, materials that meet multiple design demands could be constructed, such as waveguide filters.

A challenge in the use of the pre-trained model as conducted here is that some designs may not yield continuous solutions in 3D, which may affect mechanical stability or manufacturability. In the examples reported here, we focused specially on relatively simple cases where we achieve a continuous material design; however, variations of such scenarios can easily be constructed that fail to produce proper mechanical designs. The mechanical analysis, as depicted in Figs 7 and 13, is critical to provide a rigorous assessment of stability. In an unsupervised algorithm that explores vast spaces of designs, a rapid assessment of the mechanical properties could help to identify solutions that meet certain design demands, including mechanical stability.

The pre-trained model offers a very vast space of design solutions, far exceeding the samples considered here. Especially by combining the diffusion model with initial image cues (see Figs 10–12) provides a structurally diverse and rich platform to work from. If the existing platform is not sufficient, or if specific target designs are desirable, the model can easily be fine-tuned or retrained based on new or expanded data. This can offer not only a mechanism to use 2D image data as reported in this article but could possibly also offer a pathway to directly construct 3D data from text cues, as long as datasets exist that map conditioning constraints with resulting structural designs. This could also address inherent biases included in datasets that yield models such as Stable Diffusion, and any limitations that stem from sourcing of the data from particular sources (e.g. Internet-based text-image pairings versus more broadly researched culturally richer relationships that exist beyond the Internet). Generally, though, the framework used here can capture such richer, more diverse and larger datasets and provide ample room for improvements in a variety of forms.

The use of deep learning, and especially generative methods, opens important frontiers in materials design. The use of conditional diffusion models as used here can be expanded, or altered, to reflect materials-specific training sets. Thereby, future applications of the technology presented here can focus on models trained specifically to capture hierarchical materials, or a specific subset of bio-based material designs.

The general concepts introduced here offer many opportunities for future work, such as targeted optimization for specific material properties including mechanical deformation and fracture [68–70]. It will also be interested to further examine the image generation method and explore, systematically, the effect of variations in text prompts on the final design. Another target of study could be the exploration of variability, especially in light of recent findings that natural variability in designs seen in Nature often yield superior material performance [71–73]. Using the source of such natural variability, as predicted in the designs using Stable Diffusion, could be an interesting topic of future research.

## Supplementary Material

itac010_Supplementary_Data

## Data Availability

Data are incorporated into the article and its Online Supplementary Material. Other data are available on request.

## References

[1] Ambekar RS , Mohanty I, Kishore S et al Atomic scale structure inspired 3D-printed porous structures with tunable mechanical response. Adv Eng Mater 2021;23:2001428.

[2] Kushwaha B , Dwivedi K, Ambekar RS et al Mechanical and acoustic behavior of 3D-printed hierarchical mathematical fractal Menger sponge. Adv Eng Mater 2021;23:2001471.

[3] Ambekar RS , Kushwaha B, Sharma P et al Topologically engineered 3D printed architectures with superior mechanical strength. Mater Today 2021;48:72–94.

[4] Sajadi SM , Woellner CF, Ramesh P et al 3D printed tubulanes as lightweight hypervelocity impact resistant structures. Small 2019;15:1904747. 10.1002/smll.201904747 31709753

[5] Sajadi SM , Owuor PS, Schara S et al Multiscale geometric design principles applied to 3D printed schwarzites. Adv Mater 2018;30:1704820. 10.1002/adma.201704820 29141112

[6] Dimas LS , Buehler MJ. Influence of geometry on mechanical properties of bio-inspired silica-based hierarchical materials. Bioinspir Biomim 2012;7. https://orcid.org/10.1088/1748-3182/7/3/036024 10.1088/1748-3182/7/3/036024 22740585

[7] Mirzaeifar R , Dimas LS, Qin Z et al Defect-tolerant bioinspired hierarchical composites: Simulation and experiment. ACS Biomater Sci Eng 2015;1. https://orcid.org/10.1021/ab500120f 10.1021/ab500120f 33429576

[8] Kushwaha B , Kumar A, Ambekar RS et al Understanding the mechanics of complex topology of the 3D printed Anthill architecture. Oxford Open Mater Sci 2022;2. https://orcid.org/10.1093/OXFMAT/ITAC003

[9] Owuor PS , Hiremath S, Chipara AC et al Nature inspired strategy to enhance mechanical properties via liquid reinforcement. Adv Mater Interfaces 2017;4:1700240.

[10] Tiwary CS , Kishore S, Sarkar S et al Morphogenesis and mechanostabilization of complex natural and 3D printed shapes. Sci Adv 2015;1. https://orcid.org/10.1126/SCIADV.1400052/SUPPL_FILE/MV4.AVI 10.1126/sciadv.1400052 PMC4640649 26601170

[11] Palomba G , Hone T, Taylor D et al Bio-inspired protective structures for marine applications. Bioinspir Biomim 2020;15:056016. 32610305 10.1088/1748-3190/aba1d1

[12] Gu GX , Su I, Sharma S et al Three-dimensional-printing of bio-inspired composites. J Biomech Eng 2016;138:21006–16. 10.1115/1.4032423 PMC5101043 26747791

[13] Dimas LS , Buehler MJ. Modeling and additive manufacturing of bio-inspired composites with tunable fracture mechanical properties. Soft Matter 2014;10:4436–42. 24700202 10.1039/c3sm52890a

[14] Beese AM , Sarkar S, Nair A et al Bio-inspired carbon nanotube–polymer composite yarns with hydrogen bond-mediated lateral interactions. ACS Nano 2013;7. https://orcid.org/10.1021/nn400346r 10.1021/nn400346r 23548065

[15] Jin Y , Yuan H, le Lan J et al Bio-inspired spider-web-like membranes with a hierarchical structure for high performance lithium/sodium ion battery electrodes: the case of 3D freestanding and binder-free bismuth/CNF anodes. Nanoscale 2017;9:13298–304. 28858353 10.1039/c7nr04912a

[16] Giesa T , Spivak DI, Buehler MJ. Category theory based solution for the building block replacement problem in materials design. Adv Eng Mater 2012;14. https://orcid.org/10.1002/adem.201200109

[17] Buehler MJ. FieldPerceiver: Domain agnostic transformer model to predict multiscale physical fields and nonlinear material properties through neural ologs. Mater Today 2022;57:9–25.

[18] Spivak DI , Giesa T, Wood E et al Category theoretic analysis of hierarchical protein materials and social networks. PLoS ONE 2011;6. https://orcid.org/10.1371/journal.pone.0023911 10.1371/journal.pone.0023911 PMC3169555 21931622

[19] Ramesh A , Dhariwal P, Nichol A et al Hierarchical text-conditional image generation with CLIP latents, 2022. https://orcid.org/10.48550/arxiv.2204.06125

[20] Saharia C , Chan W, Saxena S et al Photorealistic text-to-image diffusion models with deep language understanding, 2022. https://orcid.org/10.48550/arxiv.2205.11487

[21] Rombach R , Blattmann A, Lorenz D et al High-resolution image synthesis with latent diffusion models, 2021. https://orcid.org/10.48550/arxiv.2112.10752

[22] Yeo J , Jung GS, Martin-Martinez FJ et al Materials-by-design: Computation, synthesis, and characterization from atoms to structures. Phys Scr 2018;93:53003. 10.1088/1402-4896/aab4e2 PMC6924929 31866694

[23] Buehler MJ. Materials by design—a perspective from atoms to structures. MRS Bull 2013;38:169–76. 24163499 10.1557/mrs.2013.26 PMC3806500

[24] Wegst UGK , Bai H, Saiz E et al Bioinspired structural materials. Nat Mater 2015;14:23–36. 25344782 10.1038/nmat4089

[25] Studart AR. Biological and bioinspired composites with spatially tunable heterogeneous architectures. Adv Funct Mater 2013;23:4423–36.

[26] Palkovic SD , Brommer DB, Kupwade-Patil K et al Roadmap across the mesoscale for durable and sustainable cement paste—a bioinspired approach. Constr Build Mater 2016;115:13–31.

[27] Milazzo M , Anderson GI, Buehler MJ. Bioinspired translation of classical music into de novo protein structures using deep learning and molecular modeling. Bioinspir Biomim 2021;**17**. 10.1088/1748-3190/ac338a 34700310

[28] Buehler MJ. DeepFlames: Neural network-driven self-assembly of flame particles into hierarchical structures. MRS Commun 2022;12:257–65.

[29] Yang Z , Buehler MJ. Words to matter: De novo architected materials design using transformer neural networks. Front Mater 2021;8:740754.

[30] Hsu Y-C , Yang Z, Buehler MJ. Generative design, manufacturing, and molecular modeling of 3D architected materials based on natural language input. APL Mater 2022;10:041107.

[31] Pillen A , Matthews E-K. Natural language modelled and printed in 3D: A multi-disciplinary approach. Human Soc Sci Commun 2022;9:1–11.

[32] Crowson K , Biderman S, Kornis D et al VQGAN-CLIP: Open domain image generation and editing with natural language guidance, 2022. https://orcid.org/10.48550/arxiv.2204.08583

[33] Giesa T , Spivak DI, Buehler MJ. Reoccurring patterns in hierarchical protein materials and music: The power of analogies. Bionanoscience 2011;1. https://orcid.org/10.1007/s12668-011-0022-5

[34] Giesa T , Jagadeesan R, Spivak DI et al Matriarch: A python library for materials architecture. ACS Biomater Sci Eng 2015;1. https://doi.org/10.1021/acsbiomaterials.5b00251 10.1021/acsbiomaterials.5b00251 PMC4996638 27570830

[35] Wong JY , McDonald J, Taylor-Pinney M et al Materials by design: Merging proteins and music. Nano Today 2012. https://orcid.org/10.1016/j.nantod.2012.09.001 10.1016/j.nantod.2012.09.001 PMC3752788 23997808

[36] Su I , Buehler MJ. Mesomechanics of a three-dimensional spider web. J Mech Phys Solids 2020;144. 10.1016/j.jmps.2020.104096.

[37] Su I , Narayanan N, Logrono MA et al In situ three-dimensional spider web construction and mechanics. Appl Biol Sci 2021;118:e2101296118. 10.1073/pnas.2101296118 PMC8379916 34373329

[38] Blamires SJ , Zhang S, Tso I-M. Webs: Diversity, structure and function. In: Viera C and Gonzaga M O (eds.), Behaviour and Ecology of Spiders. Cham: Springer International Publishing, 2017, 137–64.

[39] Benjamin SP , Zschokke S. Untangling the tangle-web: Web construction behavior of the comb-footed spider *Steatoda triangulosa* and comments on phylogenetic implications (Araneae: Theridiidae). J Insect Behav 2002;15:791–809.

[40] Wegst UGK. Bamboo and wood in musical instruments. Annu Rev Mater Res 2008;38:323–49.

[41] Jin K , Qin Z, Buehler MJ. Molecular deformation mechanisms of the wood cell wall material. J Mech Behav Biomed Mater 2015;42:198–206. 25498207 10.1016/j.jmbbm.2014.11.010

[42] Adler DC , Buehler MJ. Mesoscale mechanics of wood cell walls under axial strain. Soft Matter 2013;**9**:7138–44.

[43] Kabsch W , Sander C. Dictionary of protein secondary structure: Pattern recognition of hydrogen-bonded and geometrical features. Biopolymers 1983;22:2577–637. 6667333 10.1002/bip.360221211

[44] Katti MV , Sami-Subbu R, Ranjekar PK et al Amino acid repeat patterns in protein sequences: Their diversity and structural–functional implications. Protein Sci 2000;9:1203–9. 10892812 10.1110/ps.9.6.1203 PMC2144659

[45] Karras T , Aittala M, Aila T et al Elucidating the design space of diffusion-based generative models, 2022. https://orcid.org/10.48550/arxiv.2206.00364.

[46] Ho J , Jain A, Abbeel P. Denoising diffusion probabilistic models. Adv Neural Inf Process Syst 2020. https://orcid.org/10.48550/arxiv.2006.11239

[47] Dhariwal P , Nichol A. Diffusion models beat GANs on image synthesis. Adv Neural Inf Process Syst 2021;11:8780–94.

[48] Saharia C , Chan W, Chang H et al Palette: Image-to-image diffusion models, 2021. https://orcid.org/10.48550/arxiv.2111.05826

[49] Buehler MJ. Modeling atomistic dynamic fracture mechanisms using a progressive transformer diffusion model. J Appl Mech 2022;89:121009. 36389340 10.1115/1.4055730 PMC9645704

[50] Nichol A , Dhariwal P, Ramesh A et al GLIDE: Towards photorealistic image generation and editing with text-guided diffusion models, 2021. https://doi.org/10.48550/arxiv.2112.10741

[51] Buehler MJ. Predicting mechanical fields near cracks using a progressive transformer diffusion model and exploration of generalization capacity (unpublished data).

[52] Marcus G , Davis E, Aaronson S. A very preliminary analysis of DALL-E 2, 2022. https://orcid.org/10.48550/arxiv.2204.13807

[53] Wu J , Zhang C, Xue T et al Learning a probabilistic latent space of object shapes via 3D generative-adversarial modeling. In: Lee D, Sugiyama M, Luxburg U, Guyon I and Guyon R (eds.), *Advances in Neural Information Processing Systems,* 2016, 82–90.

[54] Kopanas G , Philip J, Leimkühler T et al Point-based neural rendering with per-view optimization. Comput Graph Forum 2021;40:29–43.

[55] CompVis/stable-diffusion: A latent text-to-image diffusion model. https://github.com/CompVis/stable-diffusion.

[56] Thompson AP , Aktulga HM, Berger R et al LAMMPS—a flexible simulation tool for particle-based materials modeling at the atomic, meso, and continuum scales. Comput Phys Commun 2022;271:108171.

[57] Stukowski A. Visualization and analysis of atomistic simulation data with OVITO—the open visualization tool. Model Simul Mat Sci Eng 2010;18:015012.

[58] Buehler MJ , Keten S, Ackbarow T. Theoretical and computational hierarchical nanomechanics of protein materials: Deformation and fracture. Prog Mater Sci 2008;53. https://orcid.org/10.1016/j.pmatsci.2008.06.002

[59] Breidbach O. Visions of Nature: The Art and Science of Ernst Haeckel. Munich: Prestel Verlag, 2006.

[60] Pham MS , Liu C, Todd I et al Damage-tolerant architected materials inspired by crystal microstructure. Nature 2019;565:305–11. 30651615 10.1038/s41586-018-0850-3

[61] Senhora FV , Sanders ED, Paulino GH. Optimally-tailored spinodal architected materials for multiscale design and manufacturing. Adv Mater 2022;34. https://orcid.org/10.1002/ADMA.202109304 10.1002/adma.202109304 35297113

[62] Huang W , Tarakanova A, Dinjaski N et al Design of multistimuli responsive hydrogels using integrated modeling and genetically engineered silk–elastin-like proteins. Adv Funct Mater 2016;26:4113–23. 28670244 10.1002/adfm.201600236 PMC5488272

[63] Ling S , Jin K, Qin Z et al Combining in silico design and biomimetic assembly: A new approach for developing high-performance dynamic responsive bio-nanomaterials. Adv Mater 2018;30:1802306. 10.1002/adma.201802306 PMC7189256 30260527

[64] Gu GX , Wettermark S, Buehler MJ. Algorithm-driven design of fracture resistant composite materials realized through additive manufacturing. Addit Manuf 2017;17:47–54.

[65] Gu GX , Takaffoli M, Hsieh AJ et al Biomimetic additive manufactured polymer composites for improved impact resistance. Extreme Mech Lett 2016;9. https://orcid.org/10.1016/j.eml.2016.09.006

[66] Jensen JS , Sigmund O. Systematic design of photonic crystal structures using topology optimization: Low-loss waveguide bends. Appl Phys Lett 2004;84:2022.

[67] Nielsen DG , Pedersen SD, Zhurbenko V et al Topology optimization and experimental verification of compact E-plane waveguide filters. Microw Opt Technol Lett 2019;61:1208–15.

[68] Buehler MJ , Ackbarow T. Fracture mechanics of protein materials. Mater Today 2007;10. https://orcid.org/10.1016/S1369-7021(07)70208-0

[69] Anderson TL. Fracture Mechanics: Fundamentals and Applications. Taylor & Francis, 2005.

[70] Freund LB. Dynamic fracture mechanics, 1990. https://orcid.org/10.1017/CBO9780511546761

[71] López-Valdeolivas M , Liu D, Broer DJ et al 4D printed actuators with soft-robotic functions. Macromol Rapid Commun 2018;39. https://orcid.org/10.1002/MARC.201700710 10.1002/marc.201700710 29210486

[72] Kang SH , Shan S, Noorduin WL et al Buckling-induced reversible symmetry breaking and amplification of chirality using supported cellular structures. Adv Mater 2013;25:3380–5. 23636989 10.1002/adma.201300617

[73] Li S , Librandi G, Yao Y et al Controlling liquid crystal orientations for programmable anisotropic transformations in cellular microstructures. Adv Mater 2021;33:2105024. 10.1002/adma.202105024 34473379

