# Supplementary material for: Generating 3D architectured nature-inspired materials and granular media using diffusion models based on language cues
Source: Oxf Open Mater Sci. 2022 Nov 11;2(1):itac010. doi: 10.1093/oxfmat/itac010 (PMC9767007; doi:10.1093/oxfmat/itac010)
Supplement: itac010_Supplementary_Data [file itac010_supplementary_data.pdf]

Generating 3D architectured nature-inspired materials and granular media using diffusion models based on language cues

Markus J. Buehler[1,2]*

[1] Laboratory for Atomistic and Molecular Mechanics (LAMM), Massachusetts Institute of Technology, 77 Massachusetts Ave., Cambridge, MA 02139, USA

[2] Center for Computational Science and Engineering, Schwarzman College of Computing, Massachusetts Institute of Technology, 77 Massachusetts Ave., Cambridge, MA 02139, USA

*mbuehler@MIT.EDU

SUPPLEMENTARY INFORMATION

Movies

All movies as one ZIP file: https://www.dropbox.com/s/vo3uvphu8pbtwrv/MOVIES%20M1-M6.zip?dl=0

Movie M1: Interpolation across two text prompts, generating 512 frames and rendered as a movie. https://www.dropbox.com/s/20k66xiybsr1gzj/M1.mp4?dl=0

Movie M2: High-speed recording of dropping granular media designed using the Stable Diffusion method into a glass container (10x slow-down). https://www.dropbox.com/s/q8a9srn8e8h0wu1/M2_round_drop.mp4?dl=0

Movie M3: High-speed recording of dropping granular media designed using the Stable Diffusion method into a glass container, this time for a mix of all three types of particles combine into one complex "liquid" (10x slow-down). https://www.dropbox.com/s/qjqq5f615q7jhcc/M3_mix_drop.mp4?dl=0

Movie M4: High-speed recording of dropping granular media under shaking deformation in a glass container. The liquid-like behavior can easily be confirmed (10x slow-down). https://www.dropbox.com/s/69le43elj6xtd43/M4_mix_motions.mp4?dl=0

Movie M5: Tensile deformation simulation of the material designed as described in **Figure 13.** https://www.dropbox.com/s/9chyzyc7p2lmjfe/M5.mp4?dl=0

Movie M6: Recording of the additive manufacturing process, for a variety of samples generated here. https://www.dropbox.com/s/op83t8xivzsw6rl/M6_additive_manufacturing.mp4?dl=0

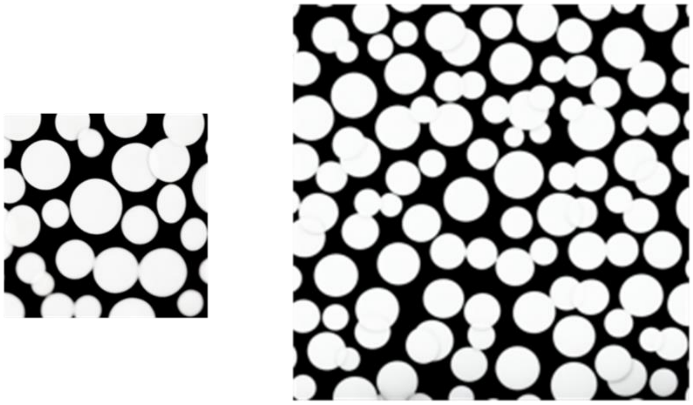

Figure S1: Comparison of generation at 512x512 pixels (left) vs. 1024x1024 pixels (right).

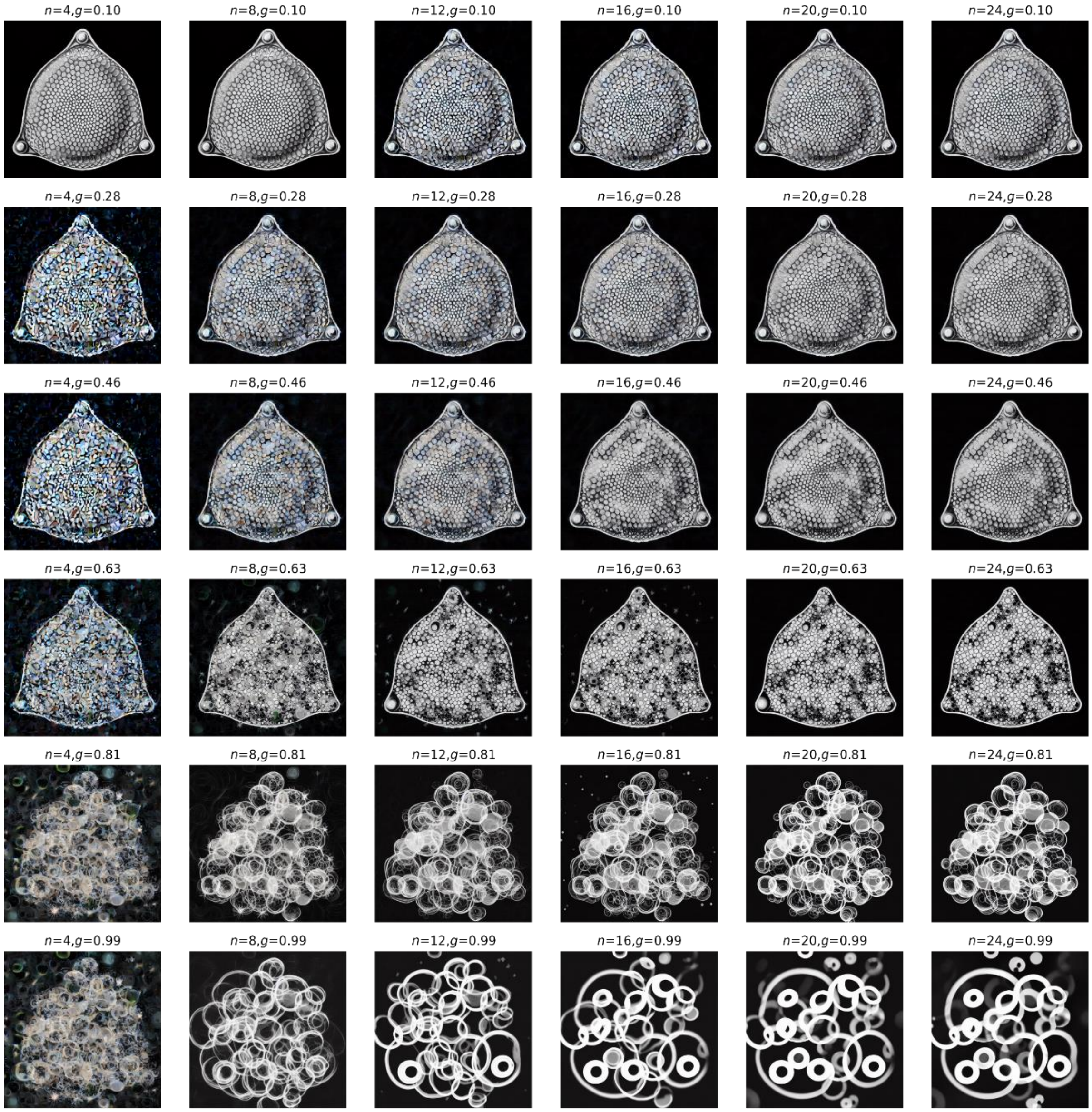

Figure S2: Variation of inference steps *n* and strength, while using the same image prompt as input as shown in **Figure 10a**. The text prompt is T_1=”several small white circles on black background". Other constant parameters $p = (S = 33)$.

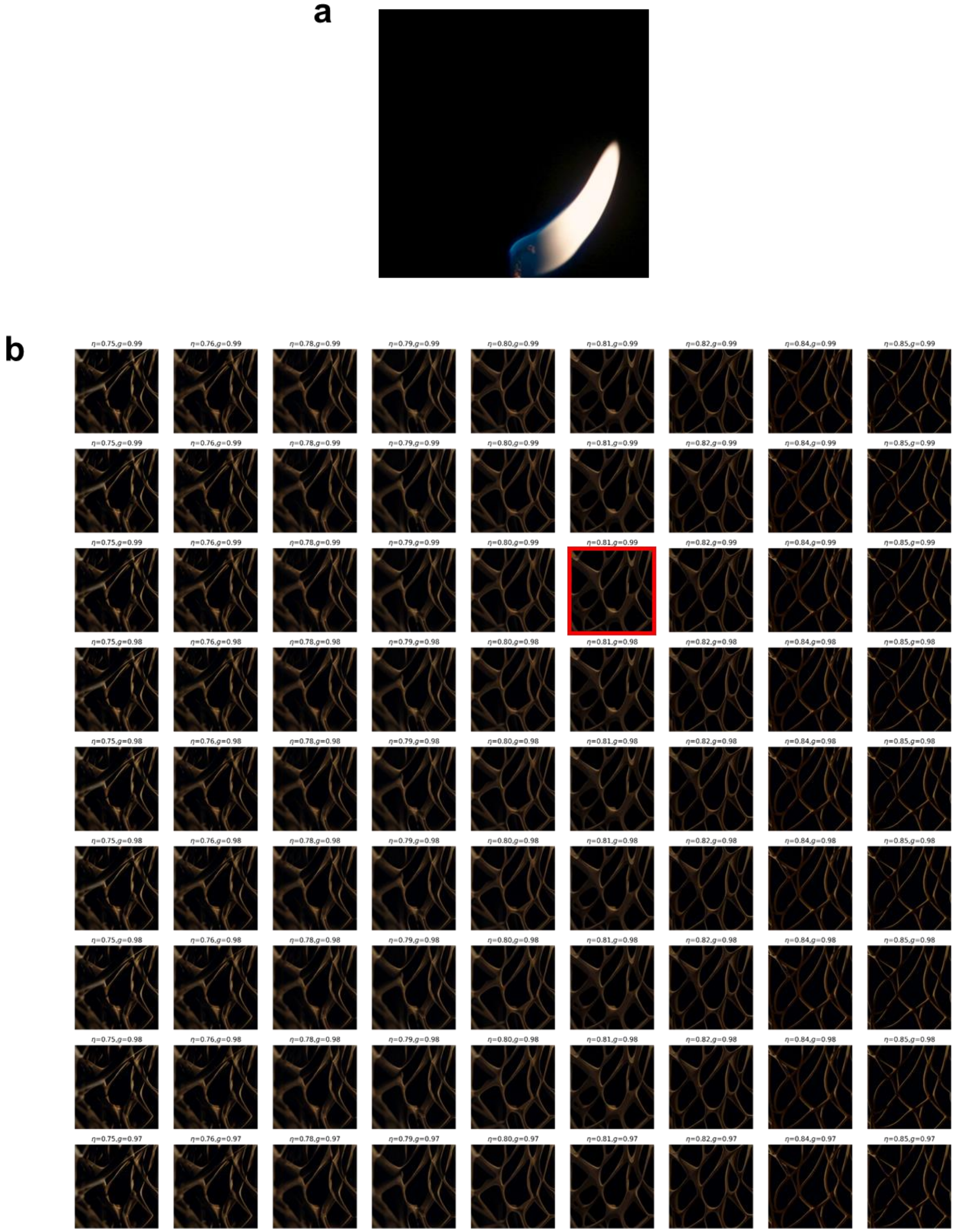

Figure S3: Candle based design. Text prompts are T_1="a spider web with thick lines on black background" and T_2="the internal details of wood microstructure". Other constant parameters $p = (S = 33, \lambda = 0.25)$.
